# Intra-aortic balloon pump (ΙΑΒΡ): from the old trends and studies to the current “extended” indications of its use

**DOI:** 10.1186/1749-8090-7-128

**Published:** 2012-12-11

**Authors:** Haralabos Parissis, Alan Soo, Bassel Al-Alao

**Affiliations:** 1 Cardiothoracic Department, Royal Victoria Hospital, Belfast BT12 6BA, Northern Ireland

## Abstract

This report outlines the well defined indications of using IABP and also favours extending the indications of IABP use, to include not only “therapeutically” the aging unstable patients but also “prophylactically” patients with low EF or high Euroscore.

## Introduction

The benefits from the IABP therapy, is due to the support of the coronary flow [1,2] and the reduction of the left ventricular load [3]. This reflects in the improvement of the oxygen supply to demand balance, aiming to reduce the size of the ischemic zone and maintain myocardial viability. This outcome leads to transient support of the left ventricular function in cases of failure during an ischemic insult. However, the effectiveness of the IABP depends on the time elapsed since the onset of the myocardial infarction, as well as on the functional stage of the left ventricle. The frequency of IABP use appears to be increasing as the proportion of high-risk patients for cardiac surgery is increasing and the complication rate is dramatically fallen to an overall rate of 6.5% and a rate of major complications requiring surgery or transfusion of 2.1% [4].

And although historically, higher complication rates have dissuaded clinicians from using it, increasing experience with favorable results during its earlier use, broadens the indications of IABP counterpulsation.

The scope of this report is to elude on to the “traditional & extended” indications of its use.

### Unstable angina not responding to medication (4% of the cases)

Although the majority of publications are not randomized studies, they still indicate that patients not responding to the maximum medical treatment can undergo surgery with stabilization through IABP with a low surgical mortality rate and low perioperative myocardial infarction rate [5,6].

Gold and associates [7] showed that the use of the intra-aortic pump eliminates the pain, improves the ST-segment elevation and prevents ventricular tachyarrhythmia. The same group showed that [8] when the IABP support, is followed by a CABG, then the outcome is statistically much better. Roberts et al. [9] agree that in unstable patients with a left ventricular dysfunction, the use of the IABP allows the safe conduct of diagnostic studies with a subsequent surgical treatment with a lower mortality.

Langou et al. [10] in 75 cases of patients where IABP was used noted a surgical mortality of 5.3% and perioperative infarction rate of 6.6%. On the contrary, a study in 55 patients with a similar presentation, operated without IABP, 14.5% died during the operation and 29% suffered a perioperative infarction.

### Supporting IABP therapy after a myocardial infarction (24.5% of the cases)

Theoretically, IABP could be used during an acute infarction in order to reduce the size and extent of the infarct, support the heart function and reduce the complications related to the incidence. The effects of IABP in 26 patients with heart failure (following an MI), were reviewed by O’Rourke et al. In the first group (n = 12 patients) ischemic pain was observed and a balloon was inserted. The second group (n = 14 patients) did not present persistent ischemic pain and the balloon was skipped. The effect on the ischemic pain was impressive: the pain stopped within a few minutes for 11 patients and within a few hours for one more patient. Out of the 14 patients in-group II, 8 in-hospital deaths occurred.

The same group of researchers [11] in a randomized clinical study, after reviewing the effect of the balloon in heart failure following a myocardial infarction, proved that there is no beneficial effect on the defined end points (morbidity, mortality). It is now accepted that in patients with acute infarction no IABP therapy is given, only as a supportive means followed by myocardial revascularization, when a cardiogenic revascularization when cardiogenic shock or any other mechanical complication that follows an infarction occurs. Since reasons for revascularization are present, the IABP method may be useful in reducing the size of the infarction and the surgical mortality [12].

In patients with acute myocardial infarction DeWood et al. [13] reported the outcomes on 40 patients who received an IABP therapy for cardiogenic shock after an infarction. Group I received IABP therapy and Group II IABP and aortocoronary bypass. Hospital mortality rates in Groups I and II were 71% and 47% respectively. The part of Group II that underwent therapy within 16 hours after the occurrence of the symptoms had lower mortality rate (25%) than that of Group II that underwent operation later than 18 hours after the symptoms were manifested (71%). Patients with acute refraction (coronary dissection or refraction due to plaque fissuring) of a minor branch of the left coronary artery due to percutaneous intervention would benefit from the IABP insertion followed by urgent revascularization [14]. Placement of an IABP in patients following myocardial infarction was most frequently indicated for cardiogenic shock (27.3%), hemodynamic support during catheterization and/or angioplasty (27.2%) or prior to high-risk surgery (11.2%), mechanical complications of acute MI (11.7%), and refractory post-myocardial infarction unstable angina (10.0%) [15].

### Supporting with IABP during the percutaneous coronary intervention (10.5% of the cases)

The effect of the IABP use during coronary catheterization is reported by Adams et al. [16] to be 1 in 1000.

However with the advent of PCI to include multivessel angioplasty, several authorities [17] have adopted the “stand-by” policy during the angioplasty in high risk patients; furthermore Balloon therapy may be the most effective treatment in the first minutes following a complicated angioplasty and as per Ferguson et al. [18] the results from the benchmark registry suggested that the most frequent indications for use of IABP was to provide hemodynamic support during or after cardiac catheterization (20.6%).

### IABP in persistent ventricular fibrillation (4.5% of the cases)

Almost all ventricular dysrhytmias attributed to ischemia can temporarily be controlled with medication, so only a few patients would need IABP insertion prior to a revascularization intervention. In patients with acute ischemia, when the ventricular fibrillation resists the ant-fibrillation treatment of second or third line, IABP [19] therapy shall be initiated and immediately after that shall follow heart catheterization and reperfusion treatment.

Patients with ventricular aneurysms and fibrillations with three-vessel disease where CABG is possible have shown good survival outcomes. However, fibrillation has remained in 30% of the cases [20], except if the aneurysm undergoes some type of surgery [21,22].

### IABP in “high risk cases” to support low cardiac output syndrome of patients undergoing cardiac surgery

In the early 70’s Berger et al. [23] and Goldman et al. [24] realized that a major indication for the IABP use is cardiac dysfunction after a cardiac intervention. They consider the possibility of urgent use of IABP after cardiac intervention, when all reasons of incomplete reperfusion have been eliminated and weaning from the cardiopulmonary device is difficult, with hypotension and low heart index despite the increased requirements of inotropic support.

In 2001, the Benchmark registry [18] shows that the IABP was used preoperatively in 13% of high-risk patients. It was then became apparent that firstly the definition of “high-risk” patient is somehow arbitrary and secondly that it is possible that preoperative IABP insertion in high-risk patients undergoing CABG may decrease mortality [25]. In a very important report from the Benchmark registry [26] the decision of instituting IABP, were compared between US and non-US centres: a larger percentage of US patients were identified as 'early' pre-operative support for high-risk CABG' (15.9% vs 6.6%). A smaller percentage of US patient’s vs non-US patients were identified as 'weaning from cardiopulmonary bypass' (14.3% vs 28.2%). In hospital mortality was lower at US vs non US sites (20.1% vs 28.7%; P < 0.001). In comparison to Benchmark, IABP at the Australian practise [27] demonstrated a prejudice toward intraoperative use (34.2% versus 16.6%; P < or = 0.0001) and an aversion to catheter laboratory support (10.6% versus 19%; P < or = 0.0001). Their outcomes demonstrate comparable mortality (22% versus 20,8; P = ns). As per Dyub et al. [28] a systematic review and meta-analysis compared a total of 1034 patients received preoperative IABP and 1329 did not receive preoperative IABP. The pooled odds ratio (OR) for hospital mortality in patients treated with preoperative IABP was 0.41 (95% CI, 0.21-0.82, p = 0.01). Preoperative IABP was associated with 3.6% absolute risk reduction in mortality and a 59% reduction in the odds for mortality in high-risk patients undergoing CABG. The evidence supports the use of preoperative IABP in high-risk patients to reduce hospital mortality. Miceli et al. [29] clearly underlying that there is no accepted consensus on the definition of high-risk patients who may benefit from the early use of intraaortic balloon pump (IABP) in coronary artery bypass grafting (CABG). They contacted an analysis in a population of 9000 patients in order to identify high-risk groups that they would potentially benefit from early IABP support. They showed that age greater than 70 years, moderate and poor left ventricular dysfunction, previous cardiac surgery, emergency operation, left main disease, Canadian Cardiovascular Society 3–4 class, and recent myocardial infarction were independent risk factors for the need of IABP insertion.

Unfortunately, the definition of high-risk population is lacking consistency in the literature and this in tern creates a bias when it comes to the criticism of all the relevant trials. Christenson and colleagues [4] defined high-risk patients as those full filling at least two of the following criteria: medically refractory unstable angina, ejection fraction less than 0.40, left main stenosis greater than 70%, and redo operation.

Another limitation of relevant reports is the lack of distinction between therapeutic for preoperative cardiogenic shock and prophylactic preoperative IABP insertion. Excluding patients receiving preoperative IABP for hemodynamic instability, shock, recent MI within 3 days, and emergency operation, Holmann and colleagues [30] found no survival advantage for use of prophylactic IABP in hemodynamically stable high-risk patients, although they showed a shorter hospital length of stay. Contrary to this a recently published propensity matched study by Lorusso et al. [31] in high-risk patients (defined as a EuroScore > 8) did in fact demonstrate a mortality benefit associated with preoperative prophylactic IABP insertion.

### Mechanical complications due to acute myocardial infarction (1.2%)

a) IABP support for acute ischemic mitral failure: Very often it involves the posterior papillary muscle, while the coronary artery responsible by 80% is the right artery. According to Wei et al., mean survival [32] without therapy is three days.

Support with IABP, surgery of the mitral valve and concomitant CABG improves survival rates [33].

b) IABP support for acute ischemic ventricular septal defect**:** In the majority of cases, cardiogenic shock with pulmonary congestion ensues. According to Logue et al. [34], the deterioration of the patient’s clinical condition depends on the extent of involvement of the right ventricle. IABP support during the ischemic ventricular septal defect increases the mean aortic pressure and the cardiac performance and reduces the right ventricular and pulmonary wedge pressure [35].

### IABP as a bridge to heart transplantation / refractory ventricular failure (7% of the cases)

Therapy with IABP reduces the meta-load improving in this way the performance of the failed heart; up to 22% of the candidates for heart transplantation [36], may require support with IABP as a bridge to transplantation.

## Conclusion

Traditionally the indications for using IABP are unstable refractory angina (4%); Supporting IABP therapy after a myocardial infarction (24.5% of the cases); catheter laboratory support (10.5%); ischemia related to intractable ventricular arrhythmias (4.5%); preoperative support (14%); weaning from cardiopulmonary bypass (34%); mechanical complications due to acute myocardial infarction (1.2%); refractory ventricular failure (7%); and other (0.3%).

The increasing early use and effectiveness of the IABP is justified and reflects the number of patients weaned successfully from the device. The success rates are higher in the high-risk groups, when the device was placed early. Therefore, beyond the traditional indications we have adopted a policy of a routine “prophylactic” preoperatively support with IABP in all patients with low ejection fraction.

However, only a prospective, randomized study in high-risk patients will really evaluate the potential merits of such a strategy.

## References

[B1] Leinbach RC Buckley MJ Austen WG Effects of intra-aortic balloon pumping on coronary flow and metabolism in man Circulation 1971 43-44 Suppl. I I-77 5317852 10.1161/01.cir.43.5s1.i-77

[B2] Swank M Singh HM Flemma RJ Effect of intra-aortic balloon pumping on nutrient coronary flow in normal and ischemic myocardium J Thorac Cardiovasc Surg 1978 76 538 703360

[B3] Berne RM Levy MN Cardiovascular physiology, ed 6, (Chap 8) 1992 St Louis: Mosby-Year Book

[B4] Cristenson JT Cohen M Ferguson JJ 3rd Freedman RJ Miller MF Ohman EM Reddy RC Stone GW Urban PM Trends in intraaortic balloon counterpulsation complications and outcomes in cardiac surgery Ann Thorac Surg 2002 74 4 1086 1090 10.1016/S0003-4975(02)03854-7 12400750

[B5] Levine FH Gold HK Leinbach RC Management of acute myocardial ischemia with intraaortic balloon pumping and coronary bypass surgery Circulation 1978 58 Suppl. I I-69 14740681

[B6] Goldman BS Gunstensten J Gilbert BW Increasing operability and survival with intra-aortic balloon pump (IABP) in cardiac surgery patients J Thorac Cardiovasc Surg 1976 72 46

[B7] Gold HK Leinbach RC Sanders CA Intra-aortic balloon pumping for control of recurrent myocardial ischemia Circulation 1973 47 1197 10.1161/01.CIR.47.6.1197 4145400

[B8] Gold HK Leinbach RC Buckley MJ Refractory angina pectons: Follow-up after intra-aortic balloon pumping and surgery Circulation 1976 54 Suppl 3 III-41 1086745

[B9] Roberts AJ Sanders JH Moran JH The efficacy of medical stabilization prior to myocardial revascularization in early refractory postinfarction angina Ann Surg 1983 197 91 6401205 PMC1352860

[B10] Langou RA Geha AS Hammond GL Cohen LS Surgical approach for patients with unstable angina pectoris: Role of the response to initial medical therapy and intraaortic balloon pumping in perioperative complications after aortocoronary bypass grafting Am J Cardiol 1978 42 629 10.1016/0002-9149(78)90633-1 100001

[B11] O'Rourke MF Norris RM Campbell TJ Randomized controlled trial of intraaortic balloon counterpulsation in early myocardial infarction with acute heart failure Am J Cardiol 1981 47 815 10.1016/0002-9149(81)90179-X 7010976

[B12] Bardet J Rigaud M Kahn JC Treatment of post-myocardial infarction angina by intra-aortic balloon pumping and emergency revascularization J Thorac Cardiovasc Surg 1977 74 299 301968

[B13] DeWood MA Notske RN Hensley GR Intraaortic balloon counterpulsation with and without reperfusion of myocardial infarction shock Circulation 1980 61 1105 10.1161/01.CIR.61.6.1105 6966191

[B14] Connors JP Phanavaro S Shaw RC Urgent myocardial revascularization for dissection of the left main coronary artery: A complication of coronary angiography J Thorac Cardiovasc Surg 1982 43 349 6981034

[B15] Stone GW Ohman EM Miller MF Joseph DL Christenson JT Contemporary utilization and outcomes of intra-aortic balloon counterpulsation in acute myocardial infarction:the benchmark registry J Am Coll Cardiol 2003 41 11 1940 1945 10.1016/S0735-1097(03)00400-5 12798561

[B16] Adams DF Fraser DB Abrams HL The complications of coronary artery arteriography Circulation 1973 48 619 10.1161/01.CIR.48.3.619 4726245

[B17] Murphy DA Craver JM Jones EL Surgical revascularization following unsuccessful percutaneous transluminal coronary angioplasty J Thorac Cardiovasc Surg 1982 84 342 6213818

[B18] Ferguson JJ 3rd Cohen M Freedman RJ Jr Stone GW Miller MF Joseph DL Ohman EM The current practice of intra-aortic balloon counterpulsation: results from the Benchmark Registry J Am Coll Cardiol 2001 38 5 1456 1462 10.1016/S0735-1097(01)01553-4 11691523

[B19] Hanson EC Levine FH Kay HR Control of postinfarction ventricular irritability with the intraaortic balloon pump Circulation 1980 62 Suppl. I I-130 7397988

[B20] Graham AF Miller C Stinson EB Surgical treatment for life-threatening ventricular arrhythmias Am. J. Cardial 1973 31 136

[B21] Culliford AT Madden MR Isom OW Glassman E Intra-aortic balloon counterpulsation. Refractory vetricular tachycardia J 1978 239 431 10.1001/jama.1978.03280320047021 621844

[B22] Harken AH Josephson ME Horowitz LN Surgical endocardial resection for the treatment of malignant ventricular tachycardia Ann Surg 1979 190 456 10.1097/00000658-197910000-00005 485619 PMC1344508

[B23] Berger RL Saini VK Ryan TJ Intra-aortic balloon assist for postcardiotomy cardiogenic shock J Thorac Cardiovasc Surg 1973 66 906 4759696

[B24] Goldman BS Walker P Gunstensen J Intra-aortic balloon pump assist: Adjunct to surgery with left ventricular dysfunction Can J Surg 1976 19 128 1260553

[B25] Field ML Rengarajan A Khan O Spyt T Richens D Preoperative intra aortic balloon pumps in patients undergoing coronary artery bypass grafting Cochrane Database Syst Rev 2007 24 CD004472 17253509 10.1002/14651858.CD004472.pub2

[B26] Cohen M Urban P Christenson JT Joseph DL Freedman RJ Jr Miller MF Benchmark Registry Collaborators. Intra-aortic balloon counterpulsation in US and non-US centres: results of the Benchmark Registry Eur Heart J 2003 24 19 1763 1770 10.1016/j.ehj.2003.07.002 14522572

[B27] Lewis PA Mullany DV Townsend S Johnson J Wood L Courtney M Joseph D Walters DL Trends in intra-aortic balloon counterpulsation: comparison of a 669 record Australian dataset with the multinational BenchmarkCounterpulsation Outcomes Registry Anaesth Intensive Care 2007 35 1 13 19 17323660 10.1177/0310057X0703500101

[B28] Dyub AM Whitlock RP Abouzahr LL Cinà CS Preoperative intra-aortic balloon pump in patients undergoing coronary bypass surgery: a systematic review and meta-analysis J Card Surg 2008 23 1 79 86 10.1111/j.1540-8191.2007.00499.x 18290898

[B29] Miceli A Duggan SM Capoun R Romeo F Caputo M Angelini GD A clinical score to predict the need for intraaortic balloon pump in patients undergoing coronary artery bypass grafting Ann Thorac Surg 2010 90 2 522 526 10.1016/j.athoracsur.2010.04.035 20667343 PMC2950276

[B30] Holmann WL Li Q Kiefe C Prophylactic value of preincision intra–aortic balloon pump: analysis of a statewide experience J Thorac Cardiovasc Surg 2000 120 1112 1119 10.1067/mtc.2000.110459 11088035

[B31] Lorusso R Gelsomino S Carella R Livi U Mariscalco G Onorati F Russo C Renzulli A Impact of prophylactic intra-aortic balloon counter-pulsation on postoperative outcome in high-risk cardiac surgery patients: a multicentre, propensity-score analysis Eur J Cardiothorac Surg 2010 38 5 585 591 10.1016/j.ejcts.2010.03.017 20399673

[B32] Wei JY Hutchins GM Buckley BH Papillary muscle rupture in fatal acute myocardial infarction. A potentially treatable form of cardiogenic shock Ann Intern Med 1979 90 149 443647 10.7326/0003-4819-90-2-149

[B33] Russo A Suri RM Grigioni F Roger VL Oh JK Mahoney DW Schaff HV Enriquez-Sarano M Clinical outcome after surgical correction of mitral regurgitation due to papillary muscle rupture Circulation 2008 118 15 1528 1534 Epub 2008 Sep 22 10.1161/CIRCULATIONAHA.107.747949 18809799

[B34] Logue B Bone D Kaplan J The diagnosis and management of mechanical defects due to myocardial infarction Cardiovasc Rev Reports 1980 1 446

[B35] Perrotta S Lentini S In patients undergoing surgical repair of post-infarction ventricular septal defect, does concomitant revascularization improve prognosis? Interact Cardiovasc Thorac Surg 2009 9 5 879 887 Epub 2009 Aug 19. Review 10.1510/icvts.2009.210658 19692439

[B36] Gjesdal O Gude E Arora S Leivestad T Andreassen AK Gullestad L Aaberge L Brunvand H Edvardsen T Geiran OR Simonsen S Intra-aortic balloon counterpulsation as a bridge to heart transplantation does not impair long-term survival Eur J Heart Fail 2009 11 7 709 714 Epub 2009 Jun 10 10.1093/eurjhf/hfp078 19515719

